# Biofilm of *Klebsiella pneumoniae* minimize phagocytosis and cytokine expression by macrophage cell line

**DOI:** 10.1186/s13568-022-01465-z

**Published:** 2022-09-19

**Authors:** Sudarshan Singh Rathore, Lalitha Cheepurupalli, Jaya Gangwar, Thiagarajan Raman, Jayapradha Ramakrishnan

**Affiliations:** 1 Centre for Research in Infectious Diseases (CRID), School of Chemical and Biotechnology (SCBT), SASTRA Deemed University, Tamil Nadu, Tirumalaisamudram, Thanjavur, 613401 India; 2 Department of Advanced Zoology and Biotechnology, Ramakrishna Mission Vivekananda College, Mylapore, Chennai, 600004 India

**Keywords:** *K. pneumoniae*, Biofilm, Phagocytosis, Macrophage, Immune response, Cytokine

## Abstract

Infectious bacteria in biofilm mode are involved in many persistent infections. Owing to its importance in clinical settings, many in vitro and in vivo studies are being conducted to study the structural and functional properties of biofilms, their drug resistant mechanism and the s urvival mechanism of planktonic and biofilm cells. In this regard, there is not sufficient information on the interaction between *Klebsiella* biofilm and macrophages. In this study, we have attempted to unravel the interaction between *Klebsiella* biofilm and macrophages in terms of phagocytic response and cytokine expression. In vitro phagocytosis assays were performed for heat inactivated and live biofilms of *K. pneumoniae,* together with the expression analysis of TLR2, iNOS, inflammatory cytokines such as IL-β1, IFN-γ, IL-6, IL-12, IL-4, TNF-α and anti-inflammatory cytokine, IL-10. A phagocytic rate of an average of 15% was observed against both heat inactivated and live biofilms when LPS + IFN-γ activated macrophages were used. This was significantly higher than non-activated macrophages when tested against heat inactivated and live biofilms (average 8%). Heat-inactivated and live biofilms induced similar phagocytic responses and up-regulation of pro-inflammatory genes in macrophages, indirectly conveying that macrophage responses are to some extent dependent on the biofilm matrix.

**Supplementary Information:**

The online version contains supplementary material available at 10.1186/s13568-022-01465-z.

## Introduction

*K. pneumoniae* is a Gram-negative, encapsulated opportunistic pathogen that colonizes almost every part of the human body with the most preferred sites being the respiratory, gastrointestinal and urinary tracts (Moore et al. 2002). *K. pneumoniae* causes both hospital and community-acquired infections (Chung 2016). Pneumonia, meningitis, urinary tract infections and catheter-related bloodstream infections are some of the potential illnesses caused by this bacterium (Szita, 2007). The major risk factors associated with *K. pneumoniae* infection include central venous catheterization, urinary catheterization, mechanical ventilation, prolonged stay in an intensive-care unit, low birth weight in preterm infants and individuals with impaired immunity (Aiola et al. 2012).

*Klebsiella* spp are characterized by the presence of capsular polysaccharides (CPS), type 1 and 3 fimbriae that act as major virulence factors and contribute to the adhesion and colonization of host tissues. Also, these virulence factors are essential for biofilm formation on indwelling medical devices (Chung 2016).

To overcome the infections caused by planktonic and biofilm forms of *K. pneumoniae*, both humoral and cell-mediated immune defenses are involved (Paczosa and Mecsas 2016). The host immune responses to planktonic *K. pneumoniae* have been extensively investigated (Blanchette and Orihuela 2012; Thurlow et al. 2011), however, information related to host immune responses to *K. pneumoniae* biofilms remain largely unexplored. The role of immune defenses and the immune evasion mechanism by *Klebsiella* sp. have been studied using both in vitro and in vivo models (Lawlor et al. 2006). And still a lack of in vivo models to study the role of biofilm development in disease pathogenesis (Guerra et al., 2022).

The first line defense mechanism includes ciliary lining, the flow of urine, peristalsis, mucus, bile and digestive enzymes (Kaper et al. 2004). Once it overcomes these mechanical barriers, humoral and cellular innate defenses function to eliminate the pathogen. Humoral defenses consist of, (i) the complement system, forms membrane attack complex and causes cell lysis, (ii) defensins, disrupt the cell membrane, and (iii) transferrin that sequesters iron. However, studies have shown that most *Klebsiella* strains appear to evade or resist the complement-mediated membrane attack formation and opsonophagocytosis under both in vitro and in vivo conditions (Paczosa and Mecsas 2016).

Besides, there have also been studies to show the importance of IL-8, CXCL1, and leukotriene B4 against infection (Paczosa and Mecsas 2016; Xiong et al. 2015). Similarly, studies on the role of TLR during *Klebsiella* infection. TLR2 plays role in lung innate immune responses and bacterial dissemination and resulting in systemic inflammation during *Klebsiella* infection (Jeon et al., 2017). Similarly, the mice defective in TLR4 signaling showed increased bacterial load and consequent mortality, suggesting the importance of bacterial recognition in immune activation (Thurlow et al. 2011). Upon bacterial recognition by pattern recognition receptors such as TLR2 and TLR4, there is an initiation of pulmonary innate immunity against *Klebsiella* by the release of cytokines such as TNF-α. Another important cytokine required for defense against *K. pneumoniae* is IL-12, which in turn is essential for the induction of IFN-γ. IFN- γ is a critical mediator in *Klebsiella* clearance and prevention of dissemination from the lungs and improved survival rates in case of localized pulmonary infection (Blanchette and Orihuela 2012). On the contrary, the detrimental role of anti-inflammatory IL-10 in persistent *K. pneumoniae* infection has also been well proved in studies (Yoshida et al. 2001). Most of these studies have looked at planktonic cells and the consequence of *K. pneumoniae* biofilm interaction with immune cells is not clear. The establishment of biofilm on the tissues of the susceptible host facilitates the expression of virulent traits and causes infections of the upper and lower respiratory tract, otitis media, tonsillitis, cystic fibrosis, urinary tract infections, and chronic bacterial prostatitis (Mahmood and Zafar 2015). Several studies in the last decade had proven the increased antibiotic resistance of *Klebsiella* in the biofilm state when compared to planktonic cells (Anderl et al. 2000; Wei et al. 2018). Recent work describes the transcriptomic profile of biofilm and dispersed cells of *K. pneumoniae* and identified signature genes as potential biomarkers of transition between biofilm and free living state (Guilhen et al., 2016).

Biofilms have also been hypothesized to contribute to reduced bacterial clearance by the innate defense mechanisms of the host. This includes limited penetration of leucocytes and their antimicrobial products into the biofilm, reduced phagocytic activity, and modified gene expression patterns and genetic shifts. Previously, our lab has explored the macrophage response toward *K. pneumoniae* planktonic cells (Lalitha et al. 2017). Further, as a first report, we have attempted to analyse the interactions between macrophage cells and *K. pneumoniae* biofilms in vitro in terms of phagocytic activity, TLR, iNOS, proinflammatory and anti-inflammatory cytokine expression during phagocytosis to understand the consequence of such interactions for persistent in vivo infections. This is based on our hypothesis that biofilms of *K. pneumoniae* can modulate macrophage responses during infection.

## Materials and methods

### Strain

The studied strain *Klebsiella pneumoniae* NDM-05–506 (MCC 2570) was procured from Microbial Culture Collection Centre, Pune, India. The strain was found to have metallo-β-lactamase, which hydrolyses almost all β-lactam antibiotics (Yong et al. 2009). The strain was further proven to be a proficient biofilm producer by our lab (Lalitha et al. 2017).

### Cell lines

Raw264.7 macrophage cell line was procured from National Centre for Cell Science, Pune, India. Cells were maintained in DMEM with 10% FBS and 1X antibiotics in 5% CO_2_ with 95% moisture at 37 °C.

### Preparation of biofilm for interaction study

To study the interactions of macrophage and *Klebsiella* biofilm, the bacteria were cultured overnight in nutrient broth at 37 °C. The culture was then centrifuged at 5000×*g* for 10 min. The cell pellets were washed with PBS and re-suspended in nutrient broth. The bacterial cells were plated at a density of 10^6^ cells/mL on acid washed coverslips (22 × 22 mm^2^), placed in 6 well plates containing nutrient broth and incubated for 72 h at 37 °C to form a biofilm. After incubation, the non-adherent cells were removed by washing thrice with sterile 1× PBS. The biofilm formed on coverslip was then transferred to 6 well plates. For phagocytosis assay, both live and heat inactivated biofilms were used. Heat inactivation was used to analyse the role of biofilm exopolysaccharide (EPS) alone in macrophage activation and immune response against *Klebsiella* biofilm. Rather than extracting the EPS, the heat method was chosen to inactivate the bacterial cells enclosed in the EPS matrix without disrupting the matrix. So where the biofilm was heat treated, it will be henceforth called heat inactivated biofilm and biofilm not given any heat treatment will be henceforth called live biofilm.

For heat inactivation, two different conditions were used: heat treatment at 56 °C for 30 min in a water bath and 56 °C for 1 h using a bacteriological incubator. After heat treatment, both live and heat inactivated biofilms were estimated by Crystal violet assay (CV).

and also microscopic assessment was made using biofilms stained with ConA-FITC (30 μg/mL) and propidium iodide (1 μg/mL).

### Phagocytosis assay

To study the phagocytic response of macrophage towards the biofilm, Raw264.7 macrophage cell line was used. Cells were harvested and placed in T25 flask in DMEM media with 10% FBS for 4 days in CO_2_ incubator with 95% humidity to get confluency. The cells were then trypsinized with trypsin–EDTA solution from T25 flask. Trypsinized macrophage cells were neutralized and stored in DMEM media with 10% FBS without antibiotics.

The cells were plated at a density of 10^5^ cells/mL with a complete DMEM medium without any antibiotics and then added to the biofilm that was formed and stained with FITC on a coverslip in 6 well plate (as mentioned in the above section). The interaction study at a ratio of 1:10 (macrophage:bacteria) was performed for both heat inactivated and live biofilm by including the following groups: (i) Non-activated macrophages added to live biofilm, (ii) non-activated macrophages added to heat inactivated biofilm for 6 h [to know the interactions between non-activated macrophages and biofilm alone], (iii) LPS + IFN-γ. activated macrophages added to live biofilm, and (iv) LPS + IFN-γ activated macrophages added to heat inactivated biofilm.

The macrophages were activated by 3 μg/mL LPS (*Pseudomonas aeruginosa* originated, L9143, Sigma) and 100 pmol IFN-γ for 6 h. [In our previous study with planktonic cells, 2 h incubation was used to activate macrophages by LPS + IFN-γ. Since we presumed that biofilm interactions need more time, we extended the time to 6 h (Lalitha et al. 2017)].

After incubation, the coverslips were washed with 1X PBS and treated with 0.005% trypan blue to quench ConA-FITC from biofilm cells (Lowe et al. 2013) that were not engulfed by macrophages. After washing, macrophage cells were stained with Giemsa stain for 15 min and then observed under a fluorescent microscope using a green filter for FITC stained biofilm, while stained macrophage was observed under bright field. The phagocytosis rate was calculated as follows:

Phagocytosis rate (%) = Macrophage with an internalised biofilm—Macrophages without internalized biofilm/Total number of macrophages × 100.

### TLR2, iNOS and cytokine expression

To analyse the expression pattern of TLR2, iNOS and cytokines in Raw 264.7 macrophages during phagocytosis of heat inactivated and live biofilm, the following experimental groups were included.

Group 1: Non-activated macrophages alone, Group 2: LPS + IFN-γ activated macrophages, Group 3: Non-activated macrophages presented to live biofilm, Group 4: LPS + IFN-γ activated macrophages presented to live biofilm, Group 5: Non-activated macrophages presented to heat inactivated biofilm, Group 6: LPS + IFN-γ activated macrophages presented to heat inactivated biofilm.

After 6 h, the cells were washed with DEPC treated 1X PBS. Total RNA was then extracted from macrophages using the TRIzol method and converted into cDNA by PrimeScript™ RT-PCR Kit (Takara). The following genes were amplified, TLR2, iNOS, proinflammatory cytokines such as IL-β1, IFN-γ, IL-6, IL-12, IL-4 and TNF-α, anti-inflammatory cytokines IL-10 and GAPDH. These genes were amplified by initial denaturation at 95 °C for 5 min followed by 40 cycles at 95 °C for 30 s. The different primers, and annealing conditions (30 s) are mentioned in Table 1 and the final extension was at 72 °C for 30 s. The primer pairs were confirmed to amplify the genomic DNA fragments of macrophages from the control group (macrophages alone). All the groups were tested in triplicate on a real time PCR system (Eppendorf, Germany) using DyNAmo Flask SYBR Green qPCR kit (Thermo Scientific) and analysed with the 2^−ΔΔCt^ method and normalized with GAPDH and control.

**Table 1:** Primer sequence used for the cytokine gene expression

Gene	Type	Sequence	Product Size (bp)	Annea-ling Temp	References
GAPDH	Forward	5′-CTGCCCAGAACATCATCCCT-3′	266	61 °C	(Stephens et al., 2011)
	Reverse	5′-GGTCCTCAGTGTAGCCCAAGA-3′			
TLR2	Forward	5′-TCGTTGTTCCCTGTGTTGCT-3′	389	65 °C	(Oshikawa and Sugiyama, 2003)
	Reverse	5′-CCACGCCCACATCATTCTCA-3′			
iNOS	Forward	5′-CACCTTGGAGTTCACCCAGT-3′	170	60 °C	(Chaturvedi et al., 2007)
	Reverse	5′-ACCACTCGTACTTGGGATGC-3′			
IL-β1	Forward	5′-ATGGCAACTGTTCCTGAACTCAACT-3′	563	60 °C	(Sjögren et al., 2002; Salgado et al., 1999)
	Reverse	5′-CAGGACAGGTATAGATTCTTTCCTTT-3′			
IFN-γ	Forward	5′-GGTTGGACAAAAAGAATCTG-3′	227	55 °C	(Munder et al., 2002)
	Reverse	5′-ACCACAGAGAGCAAGGACTT-3′			
IL-6	Forward	5′-GCCTATTGAAAATTTCCTCTG-3′	310	59 °C	(Tsoi et al., 2021)
	Reverse	5′-TAGGTTTGCCGAGTAGATCTC-3′			
IL-12	Forward	5′-CGT GCT CAT GGC TGG TGC AAA G-3′	220	61 °C	(Sisto et al., 2003)
	Reverse	5′-CTT CAT CTG CAA GTT CTT GGG C-3′			
IL-4	Forward	5′-TCG GCA TTT TGA ACG AGG TC-3′	216	57 °C	(Sisto et al., 2003)
	Reverse	5′-GGTTGGACAAAAAGAATCTG-3′			
TNF-α	Forward	5′-GAGCTTTCAACAACTACTCAG-3′	276	58 °C	(Sisto et al., 2003)
	Reverse	5′-GGAAGGCCTGAGATCTTATC-3′			
IL-10	Forward	5′-CGGGAAGACAATAACTG-3′	186	62 °C	(Sisto et al., 2003)
	Reverse	5′-CATTTCCGATAAGGCTTGG-3′			

### Statistical analysis

All experiments were performed in duplicates and the data analysis was executed in GraphPad Prism 6. Two Way ANOVA followed by Tukey's multiple comparisons test were performed to test statistical significance for multiple comparisons. Where P < 0.05 was considered to indicate a statistically significant difference. All graphs were prepared with GraphPad Prism 6 and were expressed as the mean ± SD of triplicates.

## Results

### Heat treated *K. pneumonia*e biofilms

**Fig. 1:**
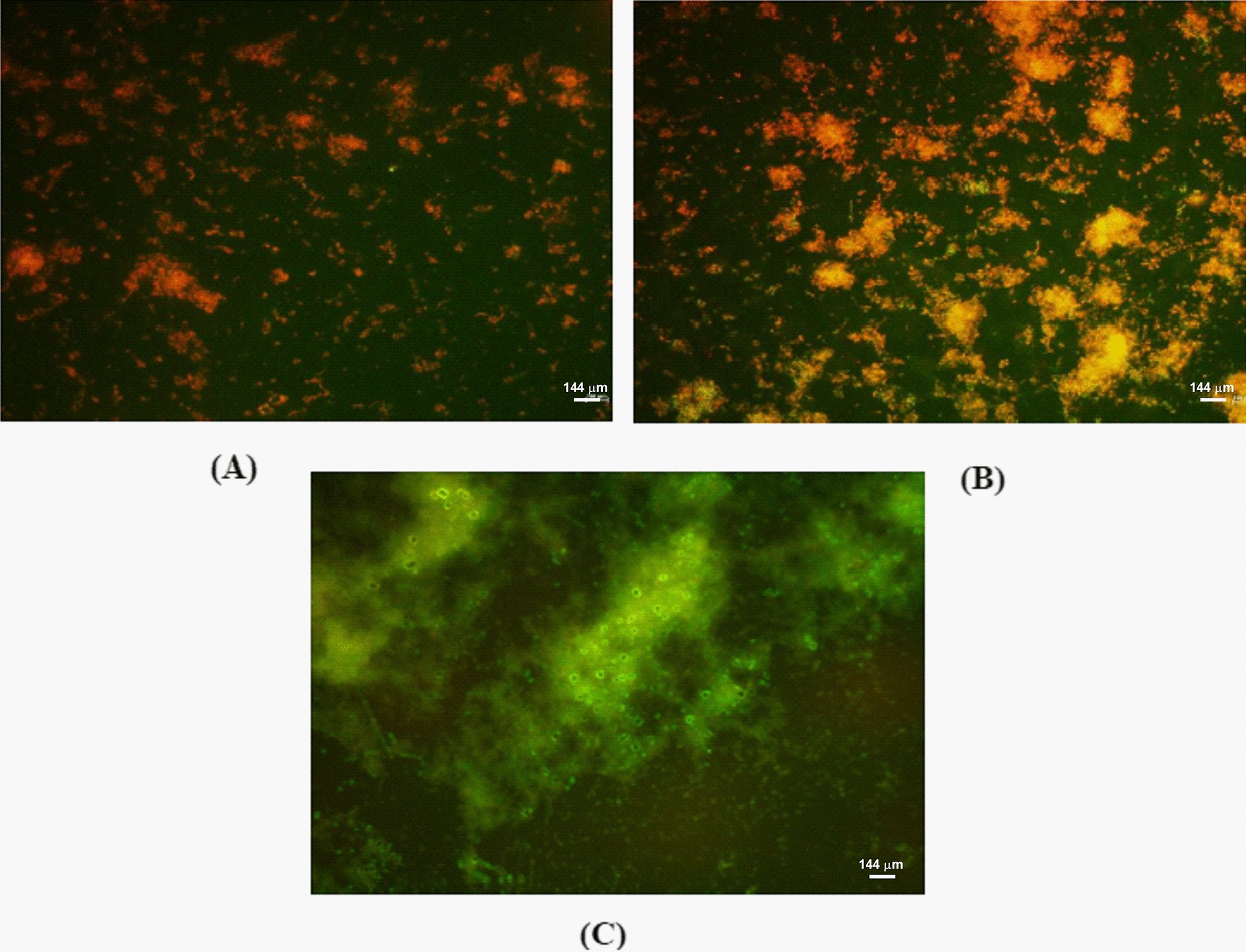
Heat-inactivated *K. pneumoniae* biofilm. **A** Heat treatment at 56 °C for 30 min in water bath; **B** 56 °C for 1 h using bacteriological incubator; **C** Untreated biofilm (Scale bar = 144 µm)

**Fig. 2:**
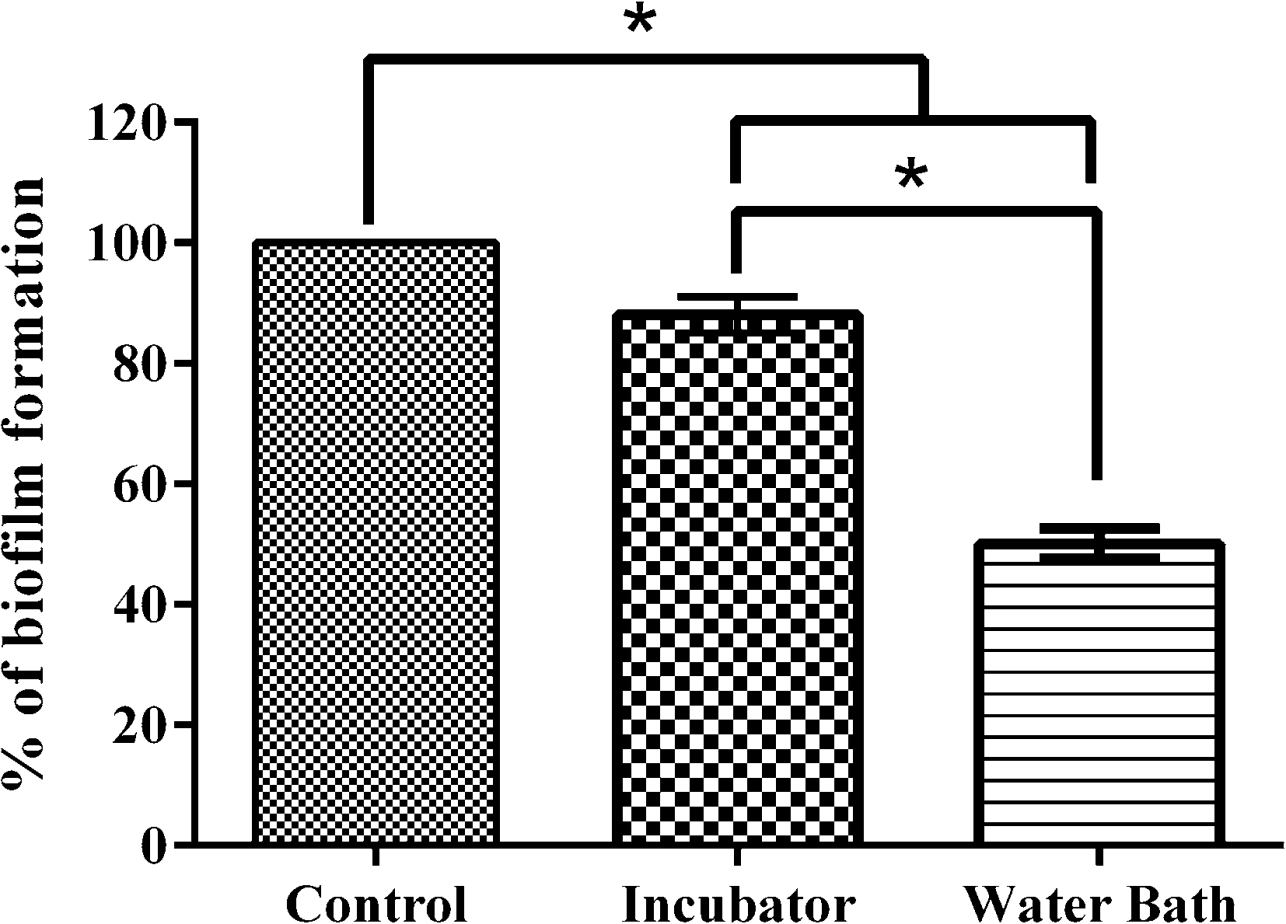
Estimation of heat-inactivated *K. pneumoniae* biofilm by CV assay. Live K. pneumoniae biofilm was inactivated by incubation at56°C for 1 h using bacteriological incubator and 56 °C for 30 min in water bath. Statistics performed by t-test, *represents significant fold increase (p < 0.05)

Figure 1, shows a visual comparison of heat-inactivated biofilms using a water bath and incubator. Dead cells were observed in both conditions. However, the number of biofilm cells were observed to be less in water bath treated when compared to incubator treated samples. The picture also reveals that the biofilm matrix was not disrupted during heat inactivation at 56 °C for 1 h using a bacteriological incubator. The untreated biofilm was observed to have dense clusters of live cells. The CV assay (Fig. 2) shows the presence of more than 80% of biofilm formation in incubated treated cells at the same time, the cells in biofilms are mostly dead which are visualised in florescent micrographs when compared with untreated biofilms. Based on these observations, biofilms inactivated using the incubator was selected for all further analyses.

### The phagocytic response of macrophage towards K. pneumoniae biofilms

**Fig. 3:**
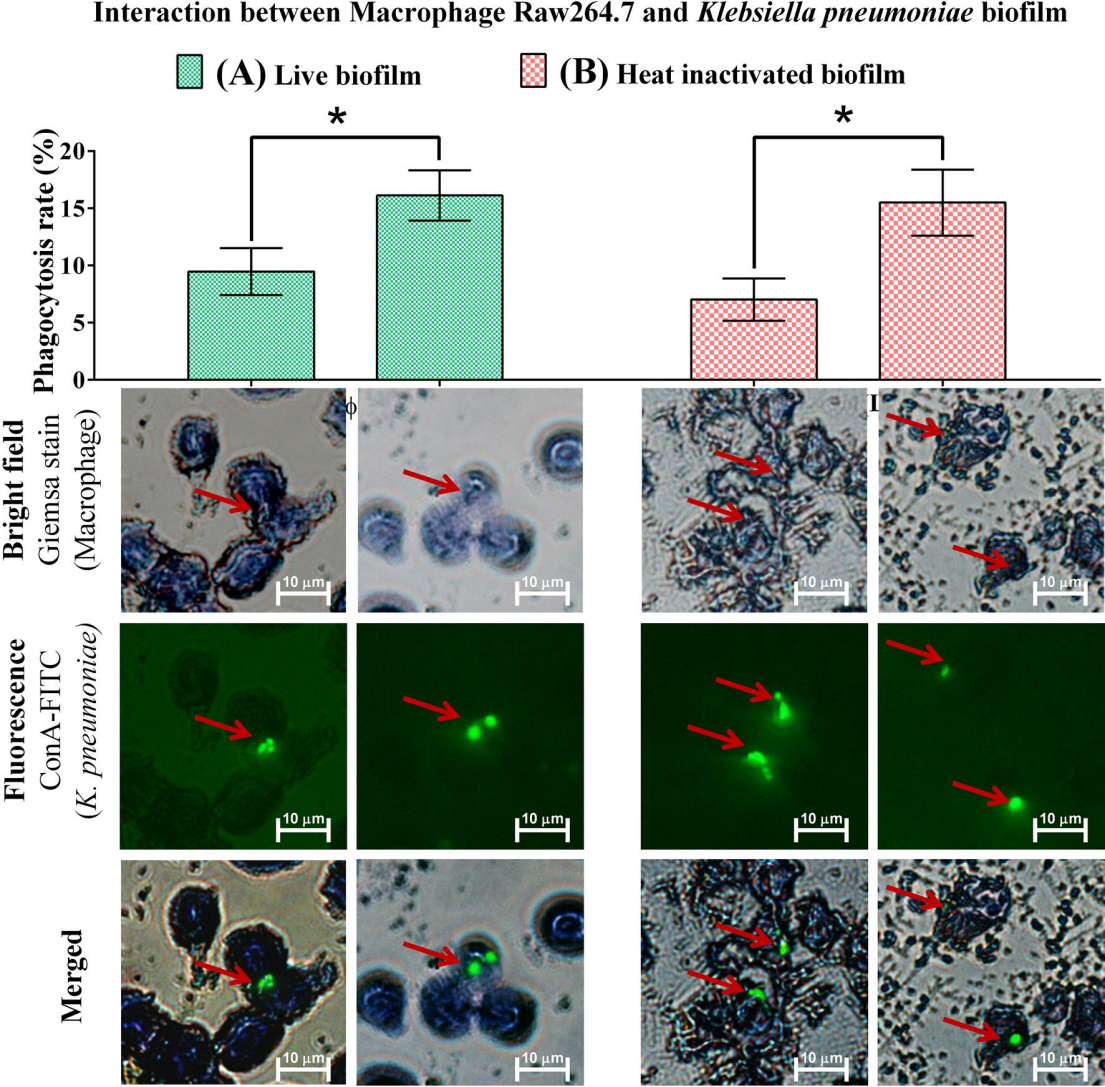
Interaction between Macrophage (Mф) Raw264.7 and *K. pneumoniae* biofilm Non- activated Mф exposed to the live biofilm (**A**) and heat inactivated biofilm (**B**); Phagocytosis assay in the presence of LPS and IFN-γ (co-treated) to the live biofilm (**A**) and Heat inactivated biofilm (**B**). Increased phagocytic response was observed in both the cases of heat inactivated and live biofilm in the presence of LPS. Where arrow represents internalized bacterial biofilm by macrophages (Scale bar = 10 µm). Statistics performed by t-test, * represents significant fold increase (p < 0.05)

To understand the role of macrophages in phagocytosis of *K. pneumoniae* biofilm, we co-incubated both these cells under different treatment conditions as mentioned earlier. LPS + IFN-γ was used to stimulate the macrophages, and heat-inactivated or live biofilms were used for interaction. As can be seen from the results (Fig. 3), the phagocytic rate of non-activated macrophage against heat inactivated biofilm was around 7 ± 1.8% as compared to that against live biofilm (9.4 ± 2.0%). This shows that there is certainly a basal response by macrophages against *K. pneumoniae* biofilms. However, when the macrophage were activated with LPS + IFN-γ before exposure to heat inactivated or live biofilms of *K. pneumoniae*, there was a significant increase in the phagocytic rate. In the case of heat inactivated biofilm, the phagocytic rate was 15 ± 2.8% when LPS + IFN-γ activated macrophages were used and in the case of live biofilm, a phagocytic rate of 16 ± 2.2% was observed when LPS + IFN-γ activated macrophages were used. It should be noted here that the presence of LPS + IFN-γ during macrophage interaction with either heat-inactivated or live biofilms consistently produced an increase in phagocytic response though it was statistically insignificant.

### Cytokine responses of RAW 264.7 macrophage to K. pneumoniae biofilms

**Fig. 4:**
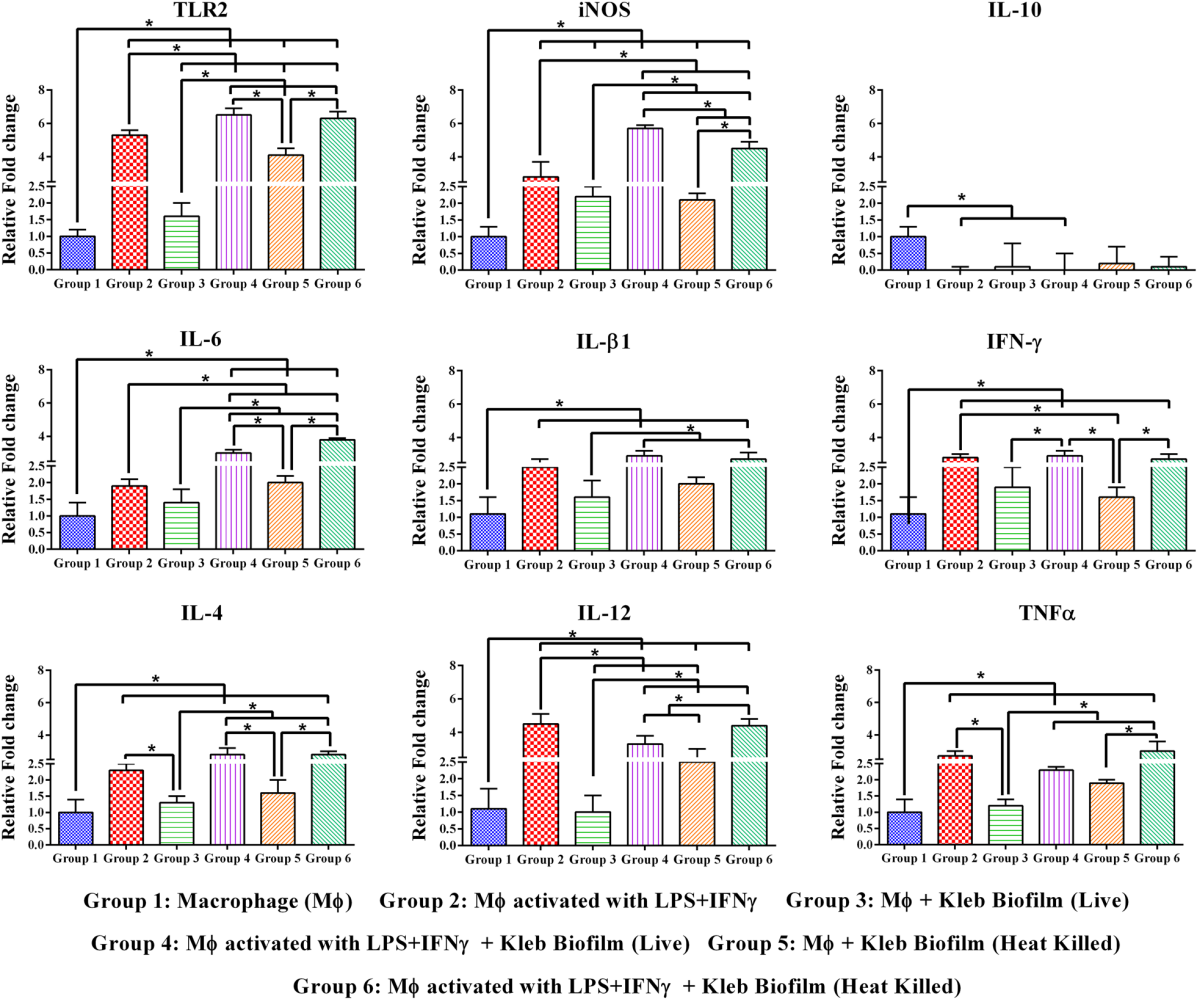
Cytokine gene expression assay. Evaluation of cytokines in RAW 264.7 Macrophage cell lines (Mф) in various conditions. Heat inactivated or live biofilms of *K. pneumoniae* exposed to activated Mф showed an increased level of cytokines(TLR2, iNOS, IL6, IL-β1, IFN-γ, IL-4, IL-12, TNF-α) except IL-10. Two Way ANOVA followed by Tukey's multiple comparisons test was performed for comparing the mean cytokine expression between the groups.*represents significant fold increase (p < 0.05)

Another aspect of macrophage-microbial interaction is the production of cytokines by macrophages as a response to both the recognition and phagocytic-killing of the microbe. To test whether this is true in the case of macrophage interaction with *K. pneumoniae* biofilms as well, we allowed LPS + IFN-γ -activated macrophages to interact with either heat-inactivated or live biofilms of *K. pneumoniae* and analysed both pro- and anti-inflammatory cytokine gene expression together with TLR2 and iNOS genes. As can be observed from the results, there was a general increase in all the pro-inflammatory cytokines (IL-β1, TNF-α, IL-6, IL-12, IFN-γ), TLR2 and iNOS when LPS was used for macrophage activation (group 2), as is well known. However, the use of LPS + IFN-γ for activation resulted in a significant increase in the anti-inflammatory IL-4 cytokine gene while there was a strong inhibition in the expression of IL-10 cytokine, when compared to unstimulated macrophages (group 1). Thus, it is clear that macrophage activation by LPS + IFN-γ is a well-recognized prerequisite for setting up of pro-inflammatory immune responses. Using this as a background data, we next introduced both LPS-activated or inactivated macrophages to either heat inactivated or live biofilms. When comparing the results of both unactivated macrophage with either heat inactivated (group 3) or live biofilm (group 5) with that of group 2 cells, it is clear that there is no significant increase in any of the genes analysed, suggesting that LPS + IFN-γ activation is very essential for inducing proinflammatory cytokine responses in macrophages. However, when group 3 responses are compared with that of group 5 cells there appears to be some change in gene expression. This is seen especially for TLR2 and IL-12 that showed a significant increase of fivefold and 1.4-fold, respectively, for group 5 cells when compared to group 3 cells (group 3 IL-12 expression was in the negative). On the other hand, group 5 macrophages showed a moderate increase in gene expression when compared to group 3 cells, while IL-10, as expected, remained unchanged (Fig. 4).

When macrophages were activated with LPS + IFN-γ and then exposed to heat inactivated (group 6) or live (group 4) biofilms there was a significant increase in all the genes analysed, except for IL-10 which showed no change. When comparing group 4 cells with group 3, it was observed that TNF-α showed the highest increase of sixfold, followed by IL-6 and IL-4 (fivefold each), iNOS (fourfold), IL-β1 (threefold) and IFN-γ (twofold). A similar but more or less consistent enhancement was observed across all the genes analysed in the case of group 6 cells when compared to group 5 macrophages, with an increase ranging from 1.7-fold to threefold. These groups of cells showed a consistent increase in gene expression when compared to group 4 macrophages. Perhaps heat treatment of biofilm modifies certain surface properties producing these differences. Nevertheless, the highest response was observed for TLR2 in the case of group 4 macrophages wherein it showed an increase of ninefold over its control (group 3) macrophage. Taken together these results suggest that (i) LPS + IFN-γ activation of macrophages is essential for a better phagocytic response towards *K. pneumoniae* biofilm and, (ii) activated macrophages can produce a stronger cytokine response when exposed to live biofilm, indicating better recognition of live biofilm cells by macrophages. (iii) Both heat-inactivated and live biofilms induced similar phagocytic responses, up regulation of pro-inflammatory genes in macrophages, indirectly conveying that macrophage response is to some extent dependent on biofilm matrix.

## Discussion

*K. pneumoniae* is a nosocomial pathogen whose resistance to antibiotics has become a problem worldwide and this is owed partly to its ability to form biofilms (Riquelme et al. 2018). Such biofilm formation is seen quite commonly with MDR strains and extensively drug resistant *K. pneumoniae* (Vuotto et al. 2017). Together with capsule and fimbriae, biofilm formation has become a major virulence mechanism (Lawlor et al. 2005) leading to immune evasion and persistent infection (Sahly et al. 2008). Although there are studies to show the immune clearance of *K. pneumoniae* strains by the cells of the immune system (Riquelme et al. 2018), the basis for such recognition and interaction is still not fully understood, and most studies have looked at only the planktonic or heat-inactivated cells. Thus, there is a general lack of information on the *K. pneumoniae* biofilm-immune cell interaction and this formed the basis of this study.

To understand the importance of biofilm in immune evasion, we used both heat-inactivated and live biofilms as targets and our results show that unactivated RAW 264.7 macrophages indeed show a basal phagocytic response towards biofilms, irrespective of the nature of the biofilm. In general, the biofilm matrix is comprised of polysaccharides, proteins, and extracellular DNA and the composition of the matrix varies with the strain of bacteria concerned (Allison 2003). We were able to obtain a higher phagocytic rate when macrophages were activated with LPS + IFN-γ, before being exposed to biofilm. Even in this case, the activated macrophages failed to distinguish between heat-inactivated and live biofilms and on average the phagocytic rate was around 15% for either heat-inactivated or live biofilms. This suggests that RAW 264.7 macrophages can phagocytose biofilm strains of *K. pneumoniae*. However, macrophage activation is needed to enhance this response, which suggests that the priming step is important for the activation of the innate immune response (Inagawa et al. 2016). Nevertheless, the rate of phagocytosis was an average of 15% for both biofilms and this could be due to a variety of factors such as incubation time, the role of biofilm in suppressing phagocytosis etc. (Riquelme et al. 2018). Similarly previous in vitro experiments demonstrated minimal *S. aureus* phagocytosis (Thurlow et al. 2011). In the case of *S. epidermidis* biofilm, impaired phagocytosis and reduced activation of J774A.1 macrophage were noticed (Schommer et al. 2011). In our previous study, we have shown the ability of the activated RAW 264.7 macrophages to phagocytose heat-inactivated planktonic *K. pneumoniae* clinical strain (Lalitha et al. 2017) by 32% and this difference we believe is due to the use of biofilm as a target here. Another criterion that could be considered for such an interaction is the exo-polysaccharide and thus it remains to be seen if macrophage activation by exo-polysaccharide of *K. pneumoniae* could be important in altering macrophage responses.

Having shown the ability of activated macrophages to phagocytose biofilms, we attempted to understand the ability of the biofilm to modulate cytokine, TLR2 and iNOS gene expression in the macrophages. It is already proven that the resistant strains of *K. pneumoniae* cannot only resist phagocytic killing but also alter the polarisation state of the macrophages (Tsuchimoto et al. 2015), which could be important in determining the successful establishment of infection by *K. pneumoniae*. A pattern of cytokine secreted is one of the major mechanisms that determine macrophage polarisation (Martinez and Gordon 2014) and microbes especially resistant ones are well known to skew the innate immune responses to a more anti-inflammatory type (Regueiro et al. 2011). Our study shows that when activated macrophages were exposed to heat-inactivated or live biofilms, there was a significant increase in pro-inflammatory cytokine genes together with the expected increase in TLR2 and iNOS. TLR-2 has been shown to be important for late stages of infection when compared to TLR-4. Interestingly one recent report by Geladari et al., 2020. (Geladari et al. 2020) have demonstrated that MNCs showed elevated TLR2, but not TLR4, response to K. pneumoniae biofilms. They have suggested that though LPS of the bacteria is important in upregulating TLR4 responses in cells, in the case of biofilms lipid A palmitoylation of LPS especially in biofilms inhibits TLR4 responses (Chalabaev et al., 2014). We believe TLR2 is an appropriate marker to understand *K. pneumoniae* infection biology.

Interestingly, anti-inflammatory IL-10 showed no up regulation in any of the treatment groups. Surprisingly, both heat-inactivated and live biofilms induced similar up regulation of pro-inflammatory genes in macrophages, suggesting a minor role of exo-polysaccharide in modulating macrophage cytokine responses. However, we would like to emphasize that this needs a more detailed study using isolated exo-polysaccharide. At this juncture, we would like to point out that in the case of group 3 macrophages (unactivated + exposed to live biofilm) and group 5 macrophages (unactivated + exposed to heat-inactivated biofilm) the gene expression was lower than in group 2 (activated macrophage alone) macrophages, suggesting the possibility of (i) suppression of macrophage immune responses by the biofilm, and (ii) the essentiality of macrophage priming by LPS + IFN-γ, at least in vitro. Nevertheless, the results of gene expression analyses clearly show that during RAW 264.7 macrophage interaction with *K. pneumoniae* biofilm, there is a modulation of the macrophage responses towards a pro-inflammatory one and this could be important in increasing the clearance efficacy of innate immune cells. Nevertheless, this modulation was not similar for LPS + IFN-γ activated Raw264.7 macrophages and planktonic *K. pneumoniae* cells The cytokine expression was found to have a significant increase in, IL-4 (eightfold), IL-12 (fivefold), TNF-α (sevenfold), IFN-γ (17-fold) (Lalitha et al. 2017), whereas, in case of biofilm, the cytokine modulation was very minimal ranging between 1 and threefold increase in cytokine expression. Taken together, the in vitro results suggest that *K. pneumoniae* in biofilm mode elicits a minimal phagocytic response and cytokine expression by macrophages.

The lack of analysing of the protein expression level using either ELISA or flow cytometry are limitations of the present study. The cytokine mRNA expression results opened up for further detailed transcriptomics approaches to understand the molecular mechanism of host pathogen interactions.

The in vitro model was developed to co-culture *Klebsiella* biofilm and macrophage cells to better understand the phagocytic response of macrophages, especially toward biofilm cells. In comparison with our previous results of macrophage and planktonic cell, response, the present study suggests that *K. pneumoniae* biofilm elicits a minimal phagocytic response and cytokine expression by macrophages. Also, these results show that for a better *K. pneumoniae* biofilm recognition and clearance by macrophages, their activation state is essential in determining both the phagocytic rate and polarisation state, at least under in vitro conditions. Perhaps this could be important in terms of devising strategies for treating extremely resistant biofilm producing strains of *K. pneumoniae*.

## Supplementary Information

**Additional file 1: Table S1.** Statistical analysis for heat inactivated biofilms. t-test was performed for the heat inactivated biofilms using an incubator and water bath (Fig. 2).

**Additional file 2: Table S2.** Statistical analysis of macrophage interactions. t-test was performed for the macrophage interactions exposed to the heat-inactivated or live biofilm (Fig. 3).

**Additional file 3: Table S3.** Statistical analysis of cytokine gene expression in Raw264.7 macrophages. Cytokines gene expression macrophage Klebsiella biofilm interaction analysis by Two Way ANOVA followed by Tukey's multiple comparisons test (TLR2, iNOS, IL-6, IL-β1, IFN-γ, IL-4, IL-12, TNF-α and IL-10) (Fig. 4).

## Data Availability

The data of this article are included within the article. For any further information on data or materials can be requested from the corresponding authors.
