# Supplementary material for: Biofilm of Klebsiella pneumoniae minimize phagocytosis and cytokine expression by macrophage cell line
Source: AMB Express. 2022 Sep 19;12:122. doi: 10.1186/s13568-022-01465-z (PMC9485320; doi:10.1186/s13568-022-01465-z)
Supplement: Supplementary file 1 — Additional file 1: Table S1. Statistical analysis for heat inactivated biofilms. t-test was performed for the heat inactivated biofilms using an incubator and water bath (Fig. 2). [file 13568_2022_1465_MOESM1_ESM.docx]

**Supplementary table 1**: t-test was performed for the heat inactivated biofilms using incubator and water bath (Figure 2).

|  | **Biofilm Formation** | | |
| --- | --- | --- | --- |
| Column B | **Incubator** | **Water Bath** | **Water Bath** |
| vs. | vs. | vs. | vs. |
| Column A | **Control** | **Incubator** | **Control** |
|  |  |  |  |
| Paired t test |  |  |  |
| P value | 0.0202 | < 0.0001 | 0.0008 |
| P value summary | * | **** | *** |
| Significantly different? (P < 0.05) | Yes | Yes | Yes |
| One- or two-tailed P value? | Two-tailed | Two-tailed | Two-tailed |
| t, df | t=6.928 df=2 | t=131.6 df=2 | t=34.64 df=2 |
| Number of pairs | 3 | 3 | 3 |
|  |  |  |  |
| How big is the difference? |  |  |  |
| Mean of differences | -12.00 | -38.00 | -50.00 |
| SD of differences | 3.000 | 0.5000 | 2.500 |
| SEM of differences | 1.732 | 0.2887 | 1.443 |
| 95% confidence interval | -19.45 to -4.548 | -39.24 to -36.76 | -56.21 to -43.79 |
| R square | 0.9600 | 0.9999 | 0.9983 |
