# Supplementary material for: Biofilm of Klebsiella pneumoniae minimize phagocytosis and cytokine expression by macrophage cell line
Source: AMB Express. 2022 Sep 19;12:122. doi: 10.1186/s13568-022-01465-z (PMC9485320; doi:10.1186/s13568-022-01465-z)
Supplement: Supplementary file 2 — Additional file 2: Table S2. Statistical analysis of macrophage interactions. t-test was performed for the macrophage interactions exposed to the heat-inactivated or live biofilm (Fig. 3). [file 13568_2022_1465_MOESM2_ESM.docx]

**Supplementary table 2**: t-test was performed for the macrophage interactions exposed to the heat-inactivated or live biofilm (Figure 3).

|  | **(A) Live Biofilm** | **(B) Heat killed biofilm** |
| --- | --- | --- |
| Column B | Mf(LPS+IFN-g) + Kp | Mf(LPS+IFN-g) + Kp |
| vs. | vs. | vs. |
| Column A | MφKp | MφKp |
|  |  |  |
| Paired t test |  |  |
| P value | 0.0002 | 0.0050 |
| P value summary | *** | ** |
| Significantly different? (P < 0.05) | Yes | Yes |
| One- or two-tailed P value? | Two-tailed | Two-tailed |
| t, df | t=79.32 df=2 | t=14.11 df=2 |
| Number of pairs | 3 | 3 |
|  |  |  |
| How big is the difference? |  |  |
| Mean of differences | 6.647 | 8.482 |
| SD of differences | 0.1452 | 1.041 |
| SEM of differences | 0.08380 | 0.6010 |
| 95% confidence interval | 6.286 to 7.008 | 5.896 to 11.07 |
| R square | 0.9997 | 0.9901 |

Where:

**Mф+ Kp:** Macrophages exposed to the *K. pneumoniae* biofilm (Live or heat inactivated);

**Mф (LPS) + Kp:** Macrophages pre treated with LPS and then exposed to *K. pneumoniae* biofilm (Live or heat inactivated).
