# Supplementary material for: Biofilm of Klebsiella pneumoniae minimize phagocytosis and cytokine expression by macrophage cell line
Source: AMB Express. 2022 Sep 19;12:122. doi: 10.1186/s13568-022-01465-z (PMC9485320; doi:10.1186/s13568-022-01465-z)
Supplement: Supplementary file 3 — Additional file 3: Table S3. Statistical analysis of cytokine gene expression in Raw264.7 macrophages. Cytokines gene expression macrophage Klebsiella biofilm interaction analysis by Two Way ANOVA followed by Tukey's multiple comparisons test (TLR2, iNOS, IL-6, IL-β1, IFN-γ, IL-4, IL-12, TNF-α and IL-10) (Fig. 4). [file 13568_2022_1465_MOESM3_ESM.docx]

**Supplementary File** 3: Cytokines gene expression macrophage Klebsiella biofilm interaction analysis by Two Way ANOVA followed by Tukey's multiple comparisons test (Figure 4).

| Two-way ANOVA | Ordinary |  |  |  |
| --- | --- | --- | --- | --- |
| Alpha | 0.05 |  |  |  |
|  |  |  |  |  |
| Source of Variation | % of total variation | P value | P value summary | Significant? |
| Interaction | 22.12 | < 0.0001 | **** | Yes |
| Row Factor | 40.11 | < 0.0001 | **** | Yes |
| Column Factor | 33.53 | < 0.0001 | **** | Yes |
|  |  |  |  |  |
| ANOVA table | SS | DF | MS | F (DFn, DFd) |
| Interaction | 86.31 | 40 | 2.158 | F (40, 108) = 14.07 |
| Row Factor | 156.5 | 8 | 19.56 | F (8, 108) = 127.6 |
| Column Factor | 130.8 | 5 | 26.16 | F (5, 108) = 170.6 |
| Residual | 16.56 | 108 | 0.1533 |  |

| Number of families | 9 |  |  |  |
| --- | --- | --- | --- | --- |
| Number of comparisons per family | 15 |  |  |  |
| Alpha | 0.05 |  |  |  |
|  |  |  |  |  |
| **Tukey's multiple comparisons test** | **Mean Diff.** | **95% CI of diff.** | **Significant?** | **Summary** |
| TLR2 |  |  |  |  |
| Group 1: Mφ vs. Group 2: Mφ activated with LPS | -4.300 | -5.228 to -3.372 | Yes | **** |
| Group 1: Mφ vs. Group 3: Mφ Kleb Biofilm | -0.6000 | -1.528 to 0.3277 | No | ns |
| Group 1: Mφ vs. Group 4: Mφ activated with LPS + Kleb Biofilm | -5.500 | -6.428 to -4.572 | Yes | **** |
| Group 1: Mφ vs. Group 5: Mφ Kleb Biofilm (HK) | -3.100 | -4.028 to -2.172 | Yes | **** |
| Group 1: Mφ vs. Group 6: Mφ activated with LPS + Kleb Biofilm (HK) | -5.300 | -6.228 to -4.372 | Yes | **** |
| Group 2: Mφ activated with LPS vs. Group 3: Mφ Kleb Biofilm | 3.700 | 2.772 to 4.628 | Yes | **** |
| Group 2: Mφ activated with LPS vs. Group 4: Mφ activated with LPS + Kleb Biofilm | -1.200 | -2.128 to -0.2723 | Yes | ** |
| Group 2: Mφ activated with LPS vs. Group 5: Mφ Kleb Biofilm (HK) | 1.200 | 0.2723 to 2.128 | Yes | ** |
| Group 2: Mφ activated with LPS vs. Group 6: Mφ activated with LPS + Kleb Biofilm (HK) | -1.000 | -1.928 to -0.07232 | Yes | * |
| Group 3: Mφ Kleb Biofilm vs. Group 4: Mφ activated with LPS + Kleb Biofilm | -4.900 | -5.828 to -3.972 | Yes | **** |
| Group 3: Mφ Kleb Biofilm vs. Group 5: Mφ Kleb Biofilm (HK) | -2.500 | -3.428 to -1.572 | Yes | **** |
| Group 3: Mφ Kleb Biofilm vs. Group 6: Mφ activated with LPS + Kleb Biofilm (HK) | -4.700 | -5.628 to -3.772 | Yes | **** |
| Group 4: Mφ activated with LPS + Kleb Biofilm vs. Group 5: Mφ Kleb Biofilm (HK) | 2.400 | 1.472 to 3.328 | Yes | **** |
| Group 4: Mφ activated with LPS + Kleb Biofilm vs. Group 6: Mφ activated with LPS + Kleb Biofilm (HK) | 0.2000 | -0.7277 to 1.128 | No | ns |
| Group 5: Mφ Kleb Biofilm (HK) vs. Group 6: Mφ activated with LPS + Kleb Biofilm (HK) | -2.200 | -3.128 to -1.272 | Yes | **** |
|  |  |  |  |  |
| IL-ß1 |  |  |  |  |
| Group 1: Mφ vs. Group 2: Mφ activated with LPS | -1.400 | -2.328 to -0.4723 | Yes | *** |
| Group 1: Mφ vs. Group 3: Mφ Kleb Biofilm | -0.5000 | -1.428 to 0.4277 | No | ns |
| Group 1: Mφ vs. Group 4: Mφ activated with LPS + Kleb Biofilm | -1.800 | -2.728 to -0.8723 | Yes | **** |
| Group 1: Mφ vs. Group 5: Mφ Kleb Biofilm (HK) | -0.9000 | -1.828 to 0.02768 | No | ns |
| Group 1: Mφ vs. Group 6: Mφ activated with LPS + Kleb Biofilm (HK) | -1.600 | -2.528 to -0.6723 | Yes | **** |
| Group 2: Mφ activated with LPS vs. Group 3: Mφ Kleb Biofilm | 0.9000 | -0.02768 to 1.828 | No | ns |
| Group 2: Mφ activated with LPS vs. Group 4: Mφ activated with LPS + Kleb Biofilm | -0.4000 | -1.328 to 0.5277 | No | ns |
| Group 2: Mφ activated with LPS vs. Group 5: Mφ Kleb Biofilm (HK) | 0.5000 | -0.4277 to 1.428 | No | ns |
| Group 2: Mφ activated with LPS vs. Group 6: Mφ activated with LPS + Kleb Biofilm (HK) | -0.2000 | -1.128 to 0.7277 | No | ns |
| Group 3: Mφ Kleb Biofilm vs. Group 4: Mφ activated with LPS + Kleb Biofilm | -1.300 | -2.228 to -0.3723 | Yes | ** |
| Group 3: Mφ Kleb Biofilm vs. Group 5: Mφ Kleb Biofilm (HK) | -0.4000 | -1.328 to 0.5277 | No | ns |
| Group 3: Mφ Kleb Biofilm vs. Group 6: Mφ activated with LPS + Kleb Biofilm (HK) | -1.100 | -2.028 to -0.1723 | Yes | * |
| Group 4: Mφ activated with LPS + Kleb Biofilm vs. Group 5: Mφ Kleb Biofilm (HK) | 0.9000 | -0.02768 to 1.828 | No | ns |
| Group 4: Mφ activated with LPS + Kleb Biofilm vs. Group 6: Mφ activated with LPS + Kleb Biofilm (HK) | 0.2000 | -0.7277 to 1.128 | No | ns |
| Group 5: Mφ Kleb Biofilm (HK) vs. Group 6: Mφ activated with LPS + Kleb Biofilm (HK) | -0.7000 | -1.628 to 0.2277 | No | ns |
|  |  |  |  |  |
| TNF-a |  |  |  |  |
| Group 1: Mφ vs. Group 2: Mφ activated with LPS | -1.700 | -2.628 to -0.7723 | Yes | **** |
| Group 1: Mφ vs. Group 3: Mφ Kleb Biofilm | -0.2000 | -1.128 to 0.7277 | No | ns |
| Group 1: Mφ vs. Group 4: Mφ activated with LPS + Kleb Biofilm | -1.300 | -2.228 to -0.3723 | Yes | ** |
| Group 1: Mφ vs. Group 5: Mφ Kleb Biofilm (HK) | -0.9000 | -1.828 to 0.02768 | No | ns |
| Group 1: Mφ vs. Group 6: Mφ activated with LPS + Kleb Biofilm (HK) | -2.000 | -2.928 to -1.072 | Yes | **** |
| Group 2: Mφ activated with LPS vs. Group 3: Mφ Kleb Biofilm | 1.500 | 0.5723 to 2.428 | Yes | *** |
| Group 2: Mφ activated with LPS vs. Group 4: Mφ activated with LPS + Kleb Biofilm | 0.4000 | -0.5277 to 1.328 | No | ns |
| Group 2: Mφ activated with LPS vs. Group 5: Mφ Kleb Biofilm (HK) | 0.8000 | -0.1277 to 1.728 | No | ns |
| Group 2: Mφ activated with LPS vs. Group 6: Mφ activated with LPS + Kleb Biofilm (HK) | -0.3000 | -1.228 to 0.6277 | No | ns |
| Group 3: Mφ Kleb Biofilm vs. Group 4: Mφ activated with LPS + Kleb Biofilm | -1.100 | -2.028 to -0.1723 | Yes | * |
| Group 3: Mφ Kleb Biofilm vs. Group 5: Mφ Kleb Biofilm (HK) | -0.7000 | -1.628 to 0.2277 | No | ns |
| Group 3: Mφ Kleb Biofilm vs. Group 6: Mφ activated with LPS + Kleb Biofilm (HK) | -1.800 | -2.728 to -0.8723 | Yes | **** |
| Group 4: Mφ activated with LPS + Kleb Biofilm vs. Group 5: Mφ Kleb Biofilm (HK) | 0.4000 | -0.5277 to 1.328 | No | ns |
| Group 4: Mφ activated with LPS + Kleb Biofilm vs. Group 6: Mφ activated with LPS + Kleb Biofilm (HK) | -0.7000 | -1.628 to 0.2277 | No | ns |
| Group 5: Mφ Kleb Biofilm (HK) vs. Group 6: Mφ activated with LPS + Kleb Biofilm (HK) | -1.100 | -2.028 to -0.1723 | Yes | * |
|  |  |  |  |  |
| IL-6 |  |  |  |  |
| Group 1: Mφ vs. Group 2: Mφ activated with LPS | -0.9000 | -1.828 to 0.02768 | No | ns |
| Group 1: Mφ vs. Group 3: Mφ Kleb Biofilm | -0.4000 | -1.328 to 0.5277 | No | ns |
| Group 1: Mφ vs. Group 4: Mφ activated with LPS + Kleb Biofilm | -2.000 | -2.928 to -1.072 | Yes | **** |
| Group 1: Mφ vs. Group 5: Mφ Kleb Biofilm (HK) | -1.000 | -1.928 to -0.07232 | Yes | * |
| Group 1: Mφ vs. Group 6: Mφ activated with LPS + Kleb Biofilm (HK) | -2.800 | -3.728 to -1.872 | Yes | **** |
| Group 2: Mφ activated with LPS vs. Group 3: Mφ Kleb Biofilm | 0.5000 | -0.4277 to 1.428 | No | ns |
| Group 2: Mφ activated with LPS vs. Group 4: Mφ activated with LPS + Kleb Biofilm | -1.100 | -2.028 to -0.1723 | Yes | * |
| Group 2: Mφ activated with LPS vs. Group 5: Mφ Kleb Biofilm (HK) | -0.1000 | -1.028 to 0.8277 | No | ns |
| Group 2: Mφ activated with LPS vs. Group 6: Mφ activated with LPS + Kleb Biofilm (HK) | -1.900 | -2.828 to -0.9723 | Yes | **** |
| Group 3: Mφ Kleb Biofilm vs. Group 4: Mφ activated with LPS + Kleb Biofilm | -1.600 | -2.528 to -0.6723 | Yes | **** |
| Group 3: Mφ Kleb Biofilm vs. Group 5: Mφ Kleb Biofilm (HK) | -0.6000 | -1.528 to 0.3277 | No | ns |
| Group 3: Mφ Kleb Biofilm vs. Group 6: Mφ activated with LPS + Kleb Biofilm (HK) | -2.400 | -3.328 to -1.472 | Yes | **** |
| Group 4: Mφ activated with LPS + Kleb Biofilm vs. Group 5: Mφ Kleb Biofilm (HK) | 1.000 | 0.07232 to 1.928 | Yes | * |
| Group 4: Mφ activated with LPS + Kleb Biofilm vs. Group 6: Mφ activated with LPS + Kleb Biofilm (HK) | -0.8000 | -1.728 to 0.1277 | No | ns |
| Group 5: Mφ Kleb Biofilm (HK) vs. Group 6: Mφ activated with LPS + Kleb Biofilm (HK) | -1.800 | -2.728 to -0.8723 | Yes | **** |
|  |  |  |  |  |
| IL-10 |  |  |  |  |
| Group 1: Mφ vs. Group 2: Mφ activated with LPS | 1.000 | 0.07232 to 1.928 | Yes | * |
| Group 1: Mφ vs. Group 3: Mφ Kleb Biofilm | 0.9000 | -0.02768 to 1.828 | No | ns |
| Group 1: Mφ vs. Group 4: Mφ activated with LPS + Kleb Biofilm | 1.000 | 0.07232 to 1.928 | Yes | * |
| Group 1: Mφ vs. Group 5: Mφ Kleb Biofilm (HK) | 0.8000 | -0.1277 to 1.728 | No | ns |
| Group 1: Mφ vs. Group 6: Mφ activated with LPS + Kleb Biofilm (HK) | 0.9000 | -0.02768 to 1.828 | No | ns |
| Group 2: Mφ activated with LPS vs. Group 3: Mφ Kleb Biofilm | -0.1000 | -1.028 to 0.8277 | No | ns |
| Group 2: Mφ activated with LPS vs. Group 4: Mφ activated with LPS + Kleb Biofilm | 0.0 | -0.9277 to 0.9277 | No | ns |
| Group 2: Mφ activated with LPS vs. Group 5: Mφ Kleb Biofilm (HK) | -0.2000 | -1.128 to 0.7277 | No | ns |
| Group 2: Mφ activated with LPS vs. Group 6: Mφ activated with LPS + Kleb Biofilm (HK) | -0.1000 | -1.028 to 0.8277 | No | ns |
| Group 3: Mφ Kleb Biofilm vs. Group 4: Mφ activated with LPS + Kleb Biofilm | 0.1000 | -0.8277 to 1.028 | No | ns |
| Group 3: Mφ Kleb Biofilm vs. Group 5: Mφ Kleb Biofilm (HK) | -0.1000 | -1.028 to 0.8277 | No | ns |
| Group 3: Mφ Kleb Biofilm vs. Group 6: Mφ activated with LPS + Kleb Biofilm (HK) | -4.967e-009 | -0.9277 to 0.9277 | No | ns |
| Group 4: Mφ activated with LPS + Kleb Biofilm vs. Group 5: Mφ Kleb Biofilm (HK) | -0.2000 | -1.128 to 0.7277 | No | ns |
| Group 4: Mφ activated with LPS + Kleb Biofilm vs. Group 6: Mφ activated with LPS + Kleb Biofilm (HK) | -0.1000 | -1.028 to 0.8277 | No | ns |
| Group 5: Mφ Kleb Biofilm (HK) vs. Group 6: Mφ activated with LPS + Kleb Biofilm (HK) | 0.1000 | -0.8277 to 1.028 | No | ns |
|  |  |  |  |  |
| IL-12 |  |  |  |  |
| Group 1: Mφ vs. Group 2: Mφ activated with LPS | -3.400 | -4.328 to -2.472 | Yes | **** |
| Group 1: Mφ vs. Group 3: Mφ Kleb Biofilm | 0.1000 | -0.8277 to 1.028 | No | ns |
| Group 1: Mφ vs. Group 4: Mφ activated with LPS + Kleb Biofilm | -2.200 | -3.128 to -1.272 | Yes | **** |
| Group 1: Mφ vs. Group 5: Mφ Kleb Biofilm (HK) | -1.400 | -2.328 to -0.4723 | Yes | *** |
| Group 1: Mφ vs. Group 6: Mφ activated with LPS + Kleb Biofilm (HK) | -3.300 | -4.228 to -2.372 | Yes | **** |
| Group 2: Mφ activated with LPS vs. Group 3: Mφ Kleb Biofilm | 3.500 | 2.572 to 4.428 | Yes | **** |
| Group 2: Mφ activated with LPS vs. Group 4: Mφ activated with LPS + Kleb Biofilm | 1.200 | 0.2723 to 2.128 | Yes | ** |
| Group 2: Mφ activated with LPS vs. Group 5: Mφ Kleb Biofilm (HK) | 2.000 | 1.072 to 2.928 | Yes | **** |
| Group 2: Mφ activated with LPS vs. Group 6: Mφ activated with LPS + Kleb Biofilm (HK) | 0.1000 | -0.8277 to 1.028 | No | ns |
| Group 3: Mφ Kleb Biofilm vs. Group 4: Mφ activated with LPS + Kleb Biofilm | -2.300 | -3.228 to -1.372 | Yes | **** |
| Group 3: Mφ Kleb Biofilm vs. Group 5: Mφ Kleb Biofilm (HK) | -1.500 | -2.428 to -0.5723 | Yes | *** |
| Group 3: Mφ Kleb Biofilm vs. Group 6: Mφ activated with LPS + Kleb Biofilm (HK) | -3.400 | -4.328 to -2.472 | Yes | **** |
| Group 4: Mφ activated with LPS + Kleb Biofilm vs. Group 5: Mφ Kleb Biofilm (HK) | 0.8000 | -0.1277 to 1.728 | No | ns |
| Group 4: Mφ activated with LPS + Kleb Biofilm vs. Group 6: Mφ activated with LPS + Kleb Biofilm (HK) | -1.100 | -2.028 to -0.1723 | Yes | * |
| Group 5: Mφ Kleb Biofilm (HK) vs. Group 6: Mφ activated with LPS + Kleb Biofilm (HK) | -1.900 | -2.828 to -0.9723 | Yes | **** |
|  |  |  |  |  |
| IL-4 |  |  |  |  |
| Group 1: Mφ vs. Group 2: Mφ activated with LPS | -1.300 | -2.228 to -0.3723 | Yes | ** |
| Group 1: Mφ vs. Group 3: Mφ Kleb Biofilm | -0.3000 | -1.228 to 0.6277 | No | ns |
| Group 1: Mφ vs. Group 4: Mφ activated with LPS + Kleb Biofilm | -1.800 | -2.728 to -0.8723 | Yes | **** |
| Group 1: Mφ vs. Group 5: Mφ Kleb Biofilm (HK) | -0.6000 | -1.528 to 0.3277 | No | ns |
| Group 1: Mφ vs. Group 6: Mφ activated with LPS + Kleb Biofilm (HK) | -1.800 | -2.728 to -0.8723 | Yes | **** |
| Group 2: Mφ activated with LPS vs. Group 3: Mφ Kleb Biofilm | 1.000 | 0.07232 to 1.928 | Yes | * |
| Group 2: Mφ activated with LPS vs. Group 4: Mφ activated with LPS + Kleb Biofilm | -0.5000 | -1.428 to 0.4277 | No | ns |
| Group 2: Mφ activated with LPS vs. Group 5: Mφ Kleb Biofilm (HK) | 0.7000 | -0.2277 to 1.628 | No | ns |
| Group 2: Mφ activated with LPS vs. Group 6: Mφ activated with LPS + Kleb Biofilm (HK) | -0.5000 | -1.428 to 0.4277 | No | ns |
| Group 3: Mφ Kleb Biofilm vs. Group 4: Mφ activated with LPS + Kleb Biofilm | -1.500 | -2.428 to -0.5723 | Yes | *** |
| Group 3: Mφ Kleb Biofilm vs. Group 5: Mφ Kleb Biofilm (HK) | -0.3000 | -1.228 to 0.6277 | No | ns |
| Group 3: Mφ Kleb Biofilm vs. Group 6: Mφ activated with LPS + Kleb Biofilm (HK) | -1.500 | -2.428 to -0.5723 | Yes | *** |
| Group 4: Mφ activated with LPS + Kleb Biofilm vs. Group 5: Mφ Kleb Biofilm (HK) | 1.200 | 0.2723 to 2.128 | Yes | ** |
| Group 4: Mφ activated with LPS + Kleb Biofilm vs. Group 6: Mφ activated with LPS + Kleb Biofilm (HK) | 7.947e-008 | -0.9277 to 0.9277 | No | ns |
| Group 5: Mφ Kleb Biofilm (HK) vs. Group 6: Mφ activated with LPS + Kleb Biofilm (HK) | -1.200 | -2.128 to -0.2723 | Yes | ** |
|  |  |  |  |  |
| IFN-g |  |  |  |  |
| Group 1: Mφ vs. Group 2: Mφ activated with LPS | -1.700 | -2.628 to -0.7723 | Yes | **** |
| Group 1: Mφ vs. Group 3: Mφ Kleb Biofilm | -0.8000 | -1.728 to 0.1277 | No | ns |
| Group 1: Mφ vs. Group 4: Mφ activated with LPS + Kleb Biofilm | -1.800 | -2.728 to -0.8723 | Yes | **** |
| Group 1: Mφ vs. Group 5: Mφ Kleb Biofilm (HK) | -0.5000 | -1.428 to 0.4277 | No | ns |
| Group 1: Mφ vs. Group 6: Mφ activated with LPS + Kleb Biofilm (HK) | -1.600 | -2.528 to -0.6723 | Yes | **** |
| Group 2: Mφ activated with LPS vs. Group 3: Mφ Kleb Biofilm | 0.9000 | -0.02768 to 1.828 | No | ns |
| Group 2: Mφ activated with LPS vs. Group 4: Mφ activated with LPS + Kleb Biofilm | -0.1000 | -1.028 to 0.8277 | No | ns |
| Group 2: Mφ activated with LPS vs. Group 5: Mφ Kleb Biofilm (HK) | 1.200 | 0.2723 to 2.128 | Yes | ** |
| Group 2: Mφ activated with LPS vs. Group 6: Mφ activated with LPS + Kleb Biofilm (HK) | 0.1000 | -0.8277 to 1.028 | No | ns |
| Group 3: Mφ Kleb Biofilm vs. Group 4: Mφ activated with LPS + Kleb Biofilm | -1.000 | -1.928 to -0.07232 | Yes | * |
| Group 3: Mφ Kleb Biofilm vs. Group 5: Mφ Kleb Biofilm (HK) | 0.3000 | -0.6277 to 1.228 | No | ns |
| Group 3: Mφ Kleb Biofilm vs. Group 6: Mφ activated with LPS + Kleb Biofilm (HK) | -0.8000 | -1.728 to 0.1277 | No | ns |
| Group 4: Mφ activated with LPS + Kleb Biofilm vs. Group 5: Mφ Kleb Biofilm (HK) | 1.300 | 0.3723 to 2.228 | Yes | ** |
| Group 4: Mφ activated with LPS + Kleb Biofilm vs. Group 6: Mφ activated with LPS + Kleb Biofilm (HK) | 0.2000 | -0.7277 to 1.128 | No | ns |
| Group 5: Mφ Kleb Biofilm (HK) vs. Group 6: Mφ activated with LPS + Kleb Biofilm (HK) | -1.100 | -2.028 to -0.1723 | Yes | * |
|  |  |  |  |  |
| iNOS |  |  |  |  |
| Group 1: Mφ vs. Group 2: Mφ activated with LPS | -1.800 | -2.728 to -0.8723 | Yes | **** |
| Group 1: Mφ vs. Group 3: Mφ Kleb Biofilm | -1.200 | -2.128 to -0.2723 | Yes | ** |
| Group 1: Mφ vs. Group 4: Mφ activated with LPS + Kleb Biofilm | -4.700 | -5.628 to -3.772 | Yes | **** |
| Group 1: Mφ vs. Group 5: Mφ Kleb Biofilm (HK) | -1.100 | -2.028 to -0.1723 | Yes | * |
| Group 1: Mφ vs. Group 6: Mφ activated with LPS + Kleb Biofilm (HK) | -3.500 | -4.428 to -2.572 | Yes | **** |
| Group 2: Mφ activated with LPS vs. Group 3: Mφ Kleb Biofilm | 0.6000 | -0.3277 to 1.528 | No | ns |
| Group 2: Mφ activated with LPS vs. Group 4: Mφ activated with LPS + Kleb Biofilm | -2.900 | -3.828 to -1.972 | Yes | **** |
| Group 2: Mφ activated with LPS vs. Group 5: Mφ Kleb Biofilm (HK) | 0.7000 | -0.2277 to 1.628 | No | ns |
| Group 2: Mφ activated with LPS vs. Group 6: Mφ activated with LPS + Kleb Biofilm (HK) | -1.700 | -2.628 to -0.7723 | Yes | **** |
| Group 3: Mφ Kleb Biofilm vs. Group 4: Mφ activated with LPS + Kleb Biofilm | -3.500 | -4.428 to -2.572 | Yes | **** |
| Group 3: Mφ Kleb Biofilm vs. Group 5: Mφ Kleb Biofilm (HK) | 0.1000 | -0.8277 to 1.028 | No | ns |
| Group 3: Mφ Kleb Biofilm vs. Group 6: Mφ activated with LPS + Kleb Biofilm (HK) | -2.300 | -3.228 to -1.372 | Yes | **** |
| Group 4: Mφ activated with LPS + Kleb Biofilm vs. Group 5: Mφ Kleb Biofilm (HK) | 3.600 | 2.672 to 4.528 | Yes | **** |
| Group 4: Mφ activated with LPS + Kleb Biofilm vs. Group 6: Mφ activated with LPS + Kleb Biofilm (HK) | 1.200 | 0.2723 to 2.128 | Yes | ** |
| Group 5: Mφ Kleb Biofilm (HK) vs. Group 6: Mφ activated with LPS + Kleb Biofilm (HK) | -2.400 | -3.328 to -1.472 | Yes | **** |
